# Protective Effects of Water Extract of *Fructus Ligustri Lucidi* against Oxidative Stress-Related Osteoporosis In Vivo and In Vitro

**DOI:** 10.3390/vetsci8090198

**Published:** 2021-09-17

**Authors:** Yi Wu, Yusheng Hu, Zeguang Zhao, Lina Xu, Ye Chen, Tongtong Liu, Qin Li

**Affiliations:** 1 Department of Veterinary Medicine, College of Life Science and Food Engineering, Hebei University of Engineering, Handan 056038, China; aimee51@163.com (Y.W.); zhaozeguang960425@163.com (Z.Z.); xulina@hebeu.edu.cn (L.X.); tncy44116@126.com (Y.C.); liu1351772106@163.com (T.L.); 2 College of Veterinary Medicine, China Agricultural University, Beijing 100193, China; hys_vet@163.com

**Keywords:** *Fructus Ligustri Lucidi*, traditional Chinese medicine, bone loss, MC3T3-E1 cells, bone mineral density, micro-CT

## Abstract

*Fructus Ligustri Lucidi* (FLL) is the fruit of *Ligustrum lucidum Ait* and is a component of many kidney-tonifying traditional Chinese medicine formulae for treating osteoporosis. Accumulating evidence has linked oxidative stress with the progression of bone diseases. The present study aimed to identify the effects of FLL on oxidative stress-related osteoporosis in vivo and in vitro. To construct animal models, we utilized d-galactose (D-gal) injection to induce oxidative stress combined with a low calcium (the exact percentage in the diet was 0.1%) diet. Thirteen-week-old Kunming female mice were gavaged with water extract of FLL for 20 days. Then, eight-month-old Kunming female mice were treated with FLL under standard administration and diet as the aged group. In vitro, MC3T3-E1 cells stimulated by H_2_O_2_ were treated with FLL for 24 h. The micro-CT results showed that the modeling approach combining oxidative stress with a low calcium diet caused low conversion type osteoporosis in mice. FLL exerted a prominent effect on preventing osteoporosis by inhibiting oxidative stress, increasing bone mineral density (BMD), improving bone microstructure, and promoting osteoblast proliferation and osteoprotegerin (OPG) protein expression; however, FLL had no therapeutic effect on bone loss in aged mice. In conclusion, FLL showed outstanding anti-bone loss ability both in vivo and in vitro and could probably be developed as a prophylactic agent for osteoporosis.

## 1. Introduction

Osteoporosis is a progressive asymptomatic bone disease characterized by a decrease in bone mass and mineral density, as well as a degeneration in the microstructure of bone tissue due to the depletion of calcium and bone protein [1]. With the development of an aging society, the prevalence and risk of osteoporosis have increased significantly and have become a public health concern, along with hypertension and diabetes [1]. Recently, oxidative stress theory has attracted increasing attention in osteoporosis research [2,3]. Studies have shown that reactive oxygen species (ROS) produced by oxidative stress disrupted the balance between bone formation-related cells, such as bone marrow mesenchymal stem cells (BMSCs), osteoblasts, osteocytes. and osteoclasts [4,5]. For instance, ROS inhibits the differentiation of bone marrow stem cells into osteoblasts and promotes the differentiation of osteoclasts, all of which lead to decreased bone mass and bone strength and ultimately aggravate osteoporosis [6]. Moreover, accelerated aging by oxidative stress increases the intracellular calcium content and reduces the calcium transmembrane distribution gradient, thereby impairing the body’s ability to absorb calcium [7]. Eventually, chronic calcium deficiency contributes to bone loss.

Existing therapeutic agents for osteoporosis are mainly anti-bone resorption drugs such as bisphosphonates, calcitonin, denosumab, and raloxifene, which have the ability to delay bone loss [8]. In addition, a few anabolic drugs containing active vitamin D and fluoride, which enhance new bone synthesis through recombinant parathyroid hormones, are also used for the treatment of osteoporosis [9]. Unfortunately, available treatments for osteoporosis have some limitations due to their limited effects on preventing bone resorption or promoting anabolism [10]. Furthermore, accumulating studies have noticed the adverse reactions of certain drugs, which restricts their long-term use [11,12].

To date, alternative therapies using natural herbal medicines that can promote bone formation and minimize side effects and bone loss have been developing [13]. In traditional Chinese medicine, *Fructus Ligustri Lucidi* (FLL), the dried fruit of *Ligustrum lucidum Ait*, is considered to be nourishment for the endocrine and renal systems and, for nearly 200 years, has been used in combination with other herbs to strengthen bone [14]. Modern investigative research has shown that over 100 compounds have been identified from FLL, including terpenoids, flavonoids, phenolic glucosides, polysaccharides, volatile components, and others, among which terpenoids are the main constituents of FLL with high content and responsible for the majority of pharmacological effects [15]. Oleanolic acid (OA) and ursolic acid (UA) are two representative triterpenoids isolated from FLL with various pharmacological activities such as antitumor, anti-inflammatory, antidiabetic, and, anticancer [16]. Notably, OA and UA have been evidenced to exhibit excellent antioxidant effects [17]. Our previous studies demonstrated that FLL and its active components could exert osteogenic effects through estrogen receptor (ER), protein kinase C (PKC), nuclear factor kappa B (NF-κB), extracellular signal-regulated kinase (ERK), jun N-terminal kinase (JNK), p38, and Akt signal transduction pathways [18]. More studies have focused on FLL promoting intestinal calcium absorption through upregulating serum 1,25(OH)_2_D_3_ and vitamin D-dependent calcium-binding protein (CaBP), thereby improving bone performance in rats [19,20]. Thus, it is of interest to know if FLL could exert the positive effects on oxidative stress-related osteoporosis in vivo and in vitro.

Most osteoporosis studies have used OVX rats as their animal models but have limitations due to differences in individual estrogen levels. Moreover, the OVX rat model takes a long time to produce estrogen-induced effects and there is only one factor responsible for such effects, i.e., estrogen. However, the pathogenesis of osteoporosis is multi-pathogenic and systemic. Hence, here, we proposed an animal model that combined oxidative stress induced by d-galactose (D-gal) injection with a low calcium diet to accelerate bone loss, which more closely resembled clinical osteoporosis caused by multiple factors in the body, including aging and calcium loss. In vitro, MC3T3-E1 cells were stimulated with H_2_O_2_ as a model of osteoblasts under oxidative stress [21]. The proliferation and differentiation of osteoblasts and the expression of osteoprotegerin protein were detected to analyze the anti-bone loss potential of FLL. The present study is designed to investigate the protective effects of FLL on oxidative stress-related osteoporosis in vivo and in vitro.

## 2. Materials and Methods

### 2.1. Chemicals and Reagents

D-galactose (D-gal) was purchased from Solarbio (Beijing, China). The culture medium, fetal bovine serum (FBS), and phosphate-buffered saline (PBS) were purchased from Thermo Fisher Scientific (Waltham, MA, USA). The penicillin and streptomycin were purchased from Hyclone (Logan, UT, USA). The Cell Counting Kit-8 (CCK-8) and the osteoprotegerin (OPG) assay kit were purchased from Dojindo Co. (Kumamoto, Japan) and R&D (Minneapolis, MN, USA), respectively. All unspecified chemicals were obtained from the Beyotime Institute of Biotechnology (Shanghai, China).

### 2.2. Preparation of Fructus Ligustri Lucidi

The raw material of *Fructus Ligustri Lucidi* (FLL) was provided by National Institutes for Food and Drug Control in Beijing. The water extract of FLL was prepared following our previous studies [18]. In short, 100 g FLL was accurately weighed and decocted twice in 1000 mL distilled water for 30 min each time, and the filtrates of the two copies were combined. Then, the supernatant was collected and concentrated under reduced pressure at 45–50 °C to make a final concentration of 1 mg/mL in water extract. After filtration and sterilization, the solution was stored at −20 °C for later use. The desired concentrations of FLL water extract were diluted with distilled water for in vivo studies and diluted with MEM-alpha culture medium for in vitro studies.

### 2.3. Animals and Animal Ethics

Forty 13-week-old female Kunming mice (average weight 42 ± 5 g) and eight 8-month-old female elder mice (average weight 45 ± 5 g) were purchased from Charles River Laboratory Animal Technology Co., Ltd. (Beijing, China). The mice were housed in a room with 12 h of light, 12 h of darkness, temperature at 22 ± 1 °C, and humidity at 55 ± 5%. All animals had free access to water and diet during the experimental period and were recorded body weight per three days. At the end of the experiment, the mice were anesthetized with sodium pentobarbital and sacrificed with cervical dislocation. The experimental protocol was approved by the Animal Ethics Committee of China Agricultural University, Beijing (no. AW41401202-2-1).

### 2.4. In Vivo Studies

#### 2.4.1. Experimental Design

To determine the effects of FLL on bone loss, 13-week-old Kunming mice (*n* = 8/group) were randomly divided as follows:

For the control group (Control), distilled water by gavage, normal saline by injection, and fed the diet with an exact Ca percentage of 1.1%; for the model group (Model), D-gal injection dose was 10 g/kg/day and dietary Ca was 0.1% (exact percentage in the diet); for the drug treatment groups, mice were treated with FLL water extract by gavage at 1.75 g/kg/d (FLL-L), 3.5 g/kg/d (FLL-M), and 7 g/kg/d (FLL-H), respectively, and were simultaneously administrated with D-gal injection and 0.1% dietary Ca.

In addition, 8-month-old Kunming mice (*n* = 8/group) were gavaged FLL at 3.5 g/kg/d (Aged) and treated with vehicle (normal saline injection and 1.1% dietary Ca).

#### 2.4.2. Sample Collection

After 20 days of treatment, all mice were housed individually in metabolic cages to collect urine and feces for 24 h. Blood from the retro-orbital sinus was collected under anesthesia before sacrifice, and the serum was prepared. Subsequently, the liver and kidneys were harvested from the mice, as well as the humerus and femurs, with soft tissues removed. All samples were stored at −80 °C for further analysis.

#### 2.4.3. Biochemical Analysis of Serum, Urine, and Fecal Samples

The concentrations of calcium (Ca) in both serum and urine samples of mice were determined by standard colorimetric methods with commercial kits (Jiancheng Ins., Nanjing, China). Creatinine (Cr) in both serum and urine samples of mice was measured by the Jaffe method with kits (Yuanmu Co., Shanghai, China). First, the feces were dried (at 110 °C for 12 h), and then ashed (at 800 °C for 12 h) in a muffle furnace. Fecal ash (100 mg) was dissolved in 2 mL of 37% HCl and diluted appropriately with Milli-Q water. The content of fecal Ca was determined by atomic absorption spectrophotometry (East & West Analytical Instruments, Beijing, China).

#### 2.4.4. Measurement of Superoxide Dismutase (SOD) and Malondialdehyde (MDA) Level of Tissues in Mice

Before analyzing, the liver and kidney tissues were ground, and the supernatant was separated. Then, the total protein content in the homogenization buffer was determined using a bicinchoninic acid (BCA) protein assay kit. The levels of superoxide dismutase (SOD) and lipid peroxidation (MDA) in the liver and kidney homogenates were evaluated with the appropriate biochemical analysis kits, according to the manufacturers’ instructions.

#### 2.4.5. Analysis of Bone Index and Bone Dry-Wet Ratio

The left humerus and femurs of mice were weighed and recorded as wet weight. Then placed them in an 80 °C incubator. After 72 h, taken out and weighed as dry weight. Bone index (mg/g) = bone wet weight/body weight × 1000; bone dry–wet ratio (%) = bone dry weight/bone wet weight × 100%.

#### 2.4.6. Micro-Computed Tomography Analysis of Mice Femurs

The metaphyseal trabecular bone of right femurs of each mouse was evaluated by micro-computed tomography (micro-CT) analysis using a scanner (SIEMENS, Munich, Germany) with the parameters as follows: Binning, l; system magnification, high; total rotation, 360 degrees; exposure time, 1500 ms; effective pixel size, 9.21 μm; real-time reconstruction; down sample factor 2. The scan reconstruction software COBRA of the micro-CT system was used to generate the original data, the three-dimensional images were generated and exported using Inveon Research Workplace (IRW, Siemens Medical Solution, Malvern, PA, USA) and Mimics software (version 14.0, Materialise Inc., Leuven, Belgium). The bone morphometric parameters calculated included bone mineral density (BMD), bone volume fraction (BV/TV), bone surface/bone volume (BS/BV), trabecular number (Tb.N), trabecular thickness (Tb.Th), trabecular separation (Tb.Sp), and trabecular bone pattern factor (Tb.Pf).

### 2.5. In Vitro Studies

#### 2.5.1. Cell Culture

The MC3T3-E1 cells were obtained from the Chinese Academy of Medical Sciences and the Institute of Basic Medical Sciences Cell Resource Center in Beijing and cultured in α-MEM containing 12% FBS and penicillin (100 U/mL)-streptomycin (100 μg/mL). These cells were incubated at 37 °C in a humidified atmosphere with 5% CO_2_. Adherent cells were collected, and the medium was changed per three days. Cells cultured in a 96-well plate were treated with H_2_O_2_ at 0.45 mM concentration and different doses (10–10^−6^ mg/mL) of FLL were added for 24 h. Cells with only α-MEM culture medium were considered to be control group (C), and thus treated with H_2_O_2_ without FLL water extract was considered to be the model group (H_2_O_2_). Each group had five duplicate wells.

#### 2.5.2. Cell Viability Assay

The effect of FLL on the cell viability of the MC3T3-E1 cells was determined using a CCK-8 assay kit. According to the manufacturer’s instructions, the optical density of each well was measured at 490 nm in a microplate analyzer (Infinite F50, TECAN, Männedorf, Switzerland). The cell proliferation rate = absorbance value of the treated cells/absorbance of the control cells × 100%.

#### 2.5.3. Detection of ALP level in Cell Supernatant

The MC3T3-E1 cells were seeded onto 96-well plates and the supernatant was collected after being cultured for 24 h. The alkaline phosphatase (ALP) level in the cell supernatant was assayed using a commercial kit and detected at a wavelength of 405 nm in a microplate analyzer (Infinite F50, TECAN, Männedorf, Switzerland).

#### 2.5.4. Expression of OPG and RANKL Protein Study

The supernatant of cells in each group was collected after being cultured for 24 h. The expression of osteoprotegerin (OPG) and receptor activator of NF-kB ligand (RANKL) were detected using the ELISA commercial kits and carried out with an enzyme label analyzer. The detection wavelength of OPG and RANKL levels were 450 nm and 405 nm, respectively.

### 2.6. Statistical Analysis

All data were presented as mean ± SD from at least three independent experiments. Statistical significance was examined by one-way analysis of variance (ANOVA) between multiple groups using SPSS software (Version 20.0, SPSS Inc., Chicago, IL, USA), and Tukey’s multiple comparison test as a post-test for comparing the group means if overall *p* < 0.05. Otherwise, Dunnett’s T3 and nonparametric tests were conducted. Statistically significant was considered to be at a value of *p* < 0.05.

## 3. Results

### 3.1. Effects of FLL on Body Weight, Serum and Urinary Biochemical, and Fecal Ca Study in Mice

**Figure 1:**
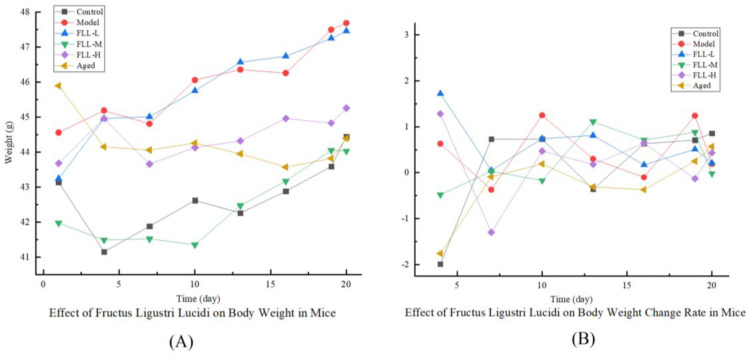
Effects of *Fructus Ligustri Lucidi* (FLL) on body weight (**A**) and weight change rate (**B**) in mice of each group. The body weight of the mice was monitored per three days throughout the study period. Weight change rate (%) = (last weight − initial weight)/initial weight × 100%.

As shown in Table 1 and Figure 1, body weights in all mice were elevated to varying degrees at the end of the experiment. Compared with the control group, the weight change rate in model mice was significantly higher (*p* < 0.01). Under FLL-L treatment, the weight gain of mice was slightly inhibited but still significantly higher than that of control mice (*p* < 0.01). FLL-M and FLL-H alleviated the trend of modeling induced weight gain in a concentration-dependent manner, with FLL-H significantly reducing (*p* < 0.05 vs. model). Unlike the model mice with increasing weight gain and maximum body weight (Figure 1), the aged mice had the lowest body weight.

Blood and urinary biochemical results (Table 1) showed that the urinary Ca/Cr ratio of model mice was obviously upregulated (*p* < 0.05, vs. control) and there was no statistical difference from aged mice. While treatment with FLL-H significantly suppressed the elevation in the urinary Ca/Cr in mice (*p* < 0.05 vs. model). Furthermore, the urinary Ca excretion was highest in the model group, but neither urinary Ca nor serum Ca of mice was altered in response to FLL treatment (Table 1). Notably, the urinary Ca in aged mice was the lowest among all groups and significantly decreased as compared with model mice (*p* < 0.01). In addition, the fecal Ca excretion in mice was prominently reduced in a dose-dependent manner by FLL treatment (*p* < 0.01 vs. model).

### 3.2. Effects of FLL on Organ Index, Bone Index, and Bone Dry–Wet Ratio

**Figure 2:**
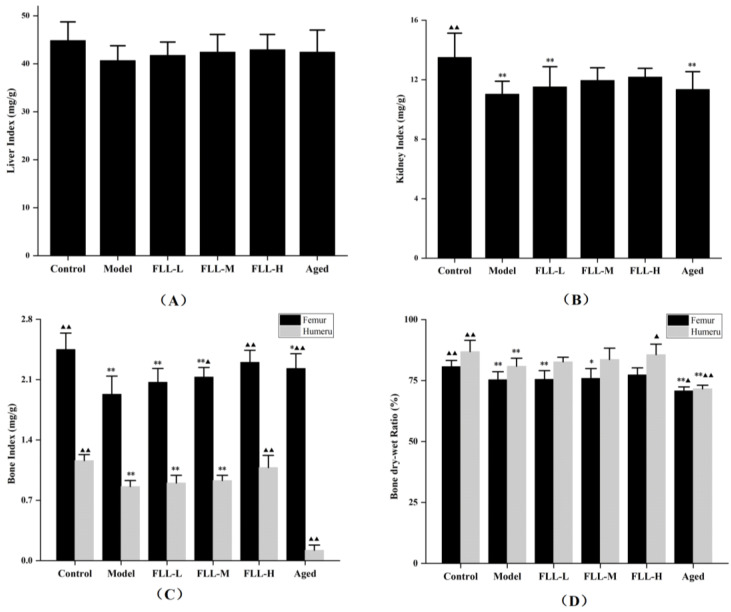
Effects of FLL on liver index (**A**), kidney index (**B**), bone index in femur and humerus (**C**), and bone dry–wet ratio in femur and humerus (**D**) in mice of each group. Organ index (mg/g) = organ weight (mg)/body weight (g). Bone index (mg/g) = bone wet weight/body weight × 1000. Bone dry–wet ratio (%) = bone dry weight/bone wet weight × 100%. Compared with the control group, * *p* < 0.05 and ** *p* < 0.01. Compared with the model group, ▲ *p* < 0.05 and ▲▲ *p* < 0.01.

Organ index showed that the kidney index of model mice was significantly reduced as compared with control mice (*p* < 0.01 vs. control, Figure 2B), but was not altered by treatment of FLL. In addition, there was no significant difference in the liver index among all mice (Figure 2A). The bone index and bone dry–wet ratio are essential indicators of bone health. As expected, the femur index and humerus index of mice were both enhanced simultaneously by FLL treatment, particularly in the FLL-H group (*p* < 0.01 vs. control, Figure 2C). FLL-M only enhanced the femur index of mice (*p* < 0.05 vs. model). The aged mice were significantly reduced either in the femoral index or humeral index as compared with the model mice (*p* < 0.01). Similarly, the dry–wet ratio of the femur and humerus in mice induced by oxidative stress combined with a low calcium diet was significantly reduced (*p* < 0.01 vs. control, Figure 2D). The FLL-H treatment observably increased the humeral dry–wet ratio in model mice (*p* < 0.05). However, there was no apparent improvement in the femoral dry–wet ratio of mice after treatment with FLL.

### 3.3. Effects of FLL on SOD Activity and MDA Content in the Liver and Kidney of Mice

**Figure 3:**
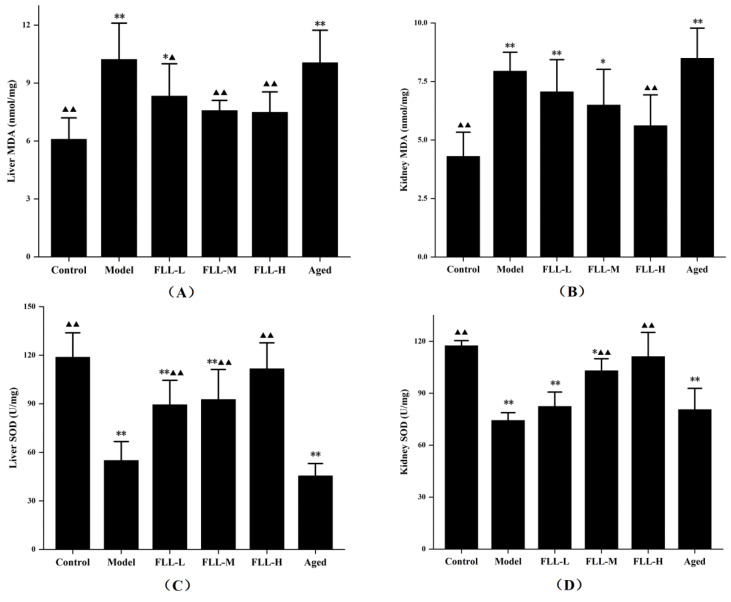
FLL treatment improved the levels of Malondialdehyde (MDA) (**A**,**B**) and Superoxide Dismutase (SOD) (**C**,**D**) in the liver and kidney of mice. The 13-week-old Kunming female mice (*n* = 8/group) and the 8-month-old aged female mice (*n* = 8) were treated with vehicle or FLL water extract at dosages of 1.75, 3.5 and 7 g/kg/d for 20 d. Meanwhile, D-gal injection (10 mg/kg/d) was combined with a low calcium diet (0.1%) to model the osteoporosis induced by oxidative stress in mice. Data are presented as mean ± SD (*n* = 8). Compared with the control group, * *p* < 0.05 and ** *p* < 0.01. Compared with the model group, ▲ *p* < 0.05 and ▲▲ *p* < 0.01.

After continuous injection of D-gal for 20 d, the MDA content in the liver and kidney of model mice was upregulated and the SOD activity was suppressed visibly (*p* < 0.01 vs. control, Figure 3A–D), indicating apparent oxidative stress injury. All concentrations of FLL improved the oxidative damage of mice to some extent, including apparently reduced MDA level (*p* < 0.01 or *p* < 0.05 vs. model, Figure 3A) and elevated SOD activity (*p* < 0.01 vs. model, Figure 3C) in the liver. Similarly, although the changes were not as evident as those in the liver, FLL also improved the oxidative status in the kidney of mice, including the MDA content was reduced and the SOD activity was increased after treatment with FLL-H (*p* < 0.01 vs. model, Figure 3B,D). Notably, no matter the MDA level or the SOD activity, the improvement of FLL-H was not statistically significant as compared with the control group. However, although the aged mice were treated with FLL-M, the MDA and SOD results in the liver and kidney did not seem to be improved noticeably. In contrast, these data were lower than that in control group (*p* < 0.01) and had no significant difference in the model group.

### 3.4. Effects of FLL on Bone Properties in Mice Analyzed by Micro-CT

**Figure 4:**
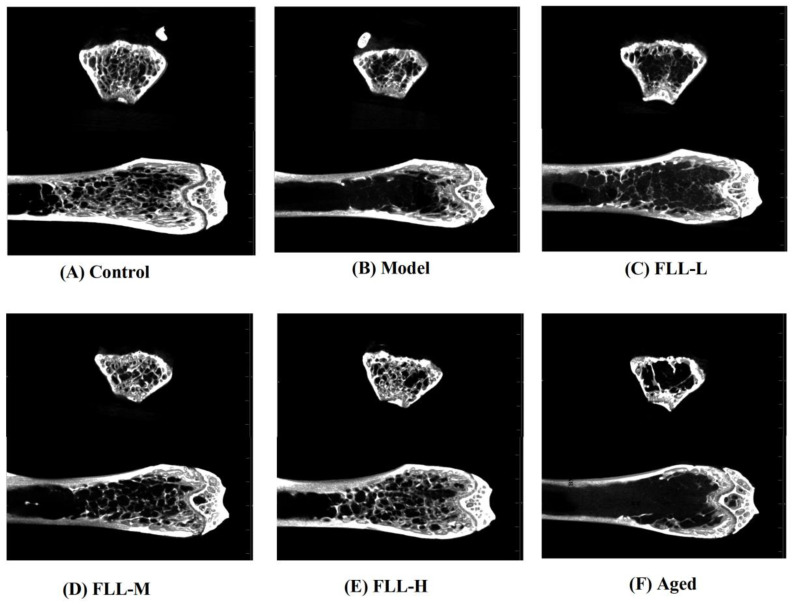
The micro-CT showed the effects of FLL on bone microstructure in the femurs of female mature (**A**–**E**) and aged mice (**F**). The 13-week-old Kunming female mice (*n* = 8/group) and 8-month-old aged female mice (*n* = 8) were orally administrated with vehicle, or FLL water extract at dosages of 1.75, 3.5 and 7 g/kg/d for 20 d. Meanwhile, D-gal injection (10 mg/kg/d) was combined with a low calcium diet (0.1%) to model the osteoporosis induced by oxidative stress in mice. At the end of the experiment, all mice were euthanized, and the right femurs were analyzed with micro-CT.

**Figure 5:**
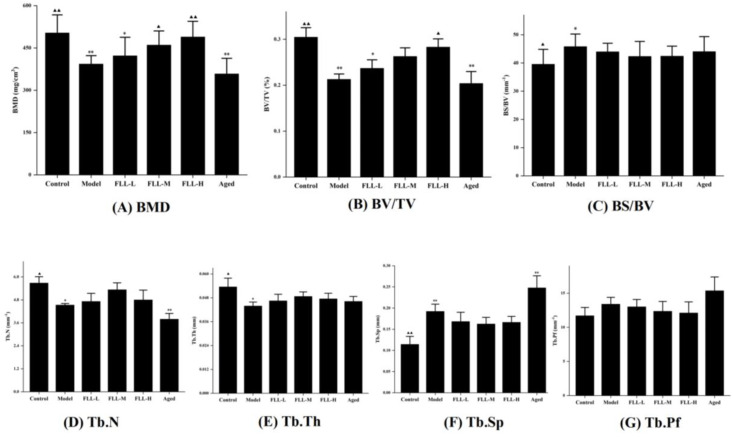
The effects of the FLL on bone trabecular parameters of the femur in osteoporosis mice analyzed by micro-CT. The trabecular analysis includes BMD (**A**), BV/TV (**B**), BS/BV (**C**), Tb.N (**D**), Tb.Th (**E**), Tb.Sp (**F**), and Tb.Pf (**G**). BMD, bone mineral density; BS/BV, specific bone surface; BV/TV, bone volume fraction; Tb.N, trabecular number; Tb.Th, trabecular thickness; Tb.Sp, trabecular separation; Tb.Pf, trabecular bone pattern factor. Data are presented as mean ± SD (*n* = 8). Compared with the control group, * *p* < 0.05 and ** *p* < 0.01. Compared with the model group, ▲ *p* < 0.05 and ▲▲ *p* < 0.01.

A low calcium diet combined with oxidative stress significantly altered the bone structure parameters of the femur in mice. The micro-CT results showed that the trabecular structure of the femur in model mice was dramatically destroyed (Figure 4B), including significantly decreased BMD, BV/TV, Tb.N, and Tb.Th, and augmented BS/BV and Tb.Sp as compared with the normal mice (*p* < 0.01 or *p* < 0.05, Figure 5A–F). Conversely, medium and high concentrations of FLL treatment prominently increased BMD (*p* < 0.05, *p* < 0.01, respectively, vs. model), and FLL-H could significantly ameliorate BV/TV in the femur of mature mice (*p* < 0.05 vs. model). We could directly find that after 20 days of continuous oral administration of FLL water extract, FLL-L, FLL-M and FLL-H groups could suppress the damage of bone trabecular structure caused by oxidative stress and low calcium diet in mature mice (Figure 4C–E). Specifically, a high concentration of FLL (Figure 4E) was found to ameliorate the trabecular bone microarchitecture extremely and seemed to restore the bone properties to almost normal levels in mice (Figure 4A). However, FLL did not reach an expected therapeutic influence on bone microarchitecture in aged mice (Figure 4F).

### 3.5. Effects of FLL on Proliferation, Differentiation and Expression of OPG and RANKL Protein in Osteoblasts under Oxidative Stress

**Figure 6:**
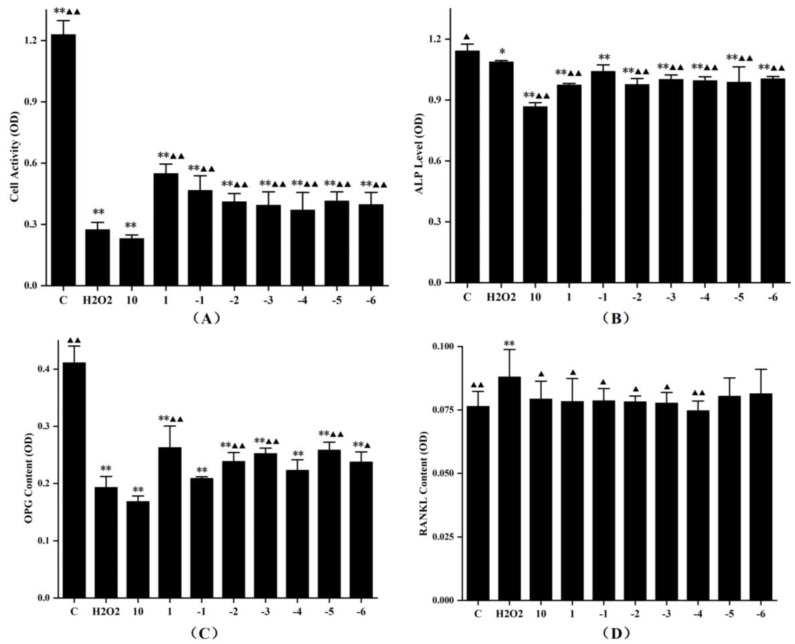
The effects of FLL water extract and 0.45 mM H_2_O_2_ on the cell viability (**A**), ALP level (**B**), and OPG protein (**C**) and RANKL protein expression (**D**) of the MC3T3-E1 cells. The cells were treated with various concentrations of FLL for 24 h. Cell viability was determined with the CCK-8 assay, and other results were detected using ELISA kits. The experiment was run twice. Data are presented as the mean ± SD, *n* = 6 for each group. * *p* < 0.05 and ** *p* < 0.01 with control group, ▲ *p* < 0.05 and ▲▲ *p* < 0.01 compared with H_2_O_2_ group.

Under the stimulation of 0.45 μM H_2_O_2_, the MC3T3-E1 cells exhibited significant oxidative stress-induced damage, as evidenced by downregulation of cell activity, ALP levels, and OPG protein expression, and upregulation of RANKL protein secretion (*p* < 0.05 vs. control, Figure 6). FLL water extract at concentrations of 1–10^−6^ mg/mL could obviously enhance the activity of cells (*p* < 0.01 vs. H_2_O_2_, Figure 6A), and their ability to promote cell proliferation was weakened as the concentrations went down in a specific range. The ALP levels of cells treated with H_2_O_2_ for 24 h were suppressed (*p* < 0.05, Figure 6B). Unexpectedly, FLL seemed to induce negative alterations on the ALP results, which were significantly downregulated at all concentrations of FLL (*p* < 0.01 vs. H_2_O_2_); there was no statistical significance among the concentration groups.

The OPG protein expression analysis indicated a dose-dependent enhancement in the cell supernatants treated with FLL water extract at a concentration gradient of 10^−1^–10^−3^ mg/mL or 10^−4^–10^−5^ mg/mL. Moreover, the highest content of OPG protein was observed when the concentration of FLL was 1 mg/mL (*p* < 0.01, vs. H_2_O_2_; Figure 6C). H_2_O_2_ up-regulated the RANKL protein expression in osteoblasts (*p* < 0.01, vs. C; Figure 6D), whereas FLL at 10–10^−4^ mg/mL significantly down-regulated the RANKL protein secretion under oxidative stress (*p* < 0.01 or *p* < 0.05, vs. H_2_O_2_). In addition, 10 mg/mL FLL suppressed cell activity, ALP level (*p* < 0.05, vs. H_2_O_2_) and OPG protein secretion, and promoted the RANKL protein expression.

## 4. Discussion

The reactive oxygen species (ROS) was inevitably produced in the process of organism metabolism. When the balance between production and elimination of ROS in the body was disturbed, such as in aging and diseases, oxidative stress occurred [5]. Excessive injection of d-galactose (D-gal) elevated the concentration of reducing aldose in the body. Accordingly, the galactitol, which cannot be adequately metabolized, accumulated in the body and caused cell swelling and death [22]. Simultaneously, large amounts of free radicals produced during metabolism contributed to cellular damage and dysfunction, ultimately accelerating oxidative stress and aging in the body [23]. After modeling, the malondialdehyde (MDA) contents in the liver and kidney of mice increased and superoxide dismutase (SOD) activity decreased, which were evident manifestations of oxidative damage (*p* < 0.01, Figure 3). Conversely, *Fructus Ligustri Lucidi* (FLL) exhibited antioxidant activity by reducing MDA levels and elevating SOD activity in a dose-dependent manner (*p* < 0.01 or *p* < 0.05). Moreover, rapid weight gain was also a characteristic of metabolic disorders associated with D-gal [24]. Our in vivo studies showed a significant weight gain in mature mice after D-gal injection for 20 days. FLL ameliorated abnormal weight gain trends by inhibiting oxidative stress (*p* < 0.01, Table 1). The present study did not obtain statistical changes in the organ index of mice, while the growth curve and the weight change rate both indicated that FLL could significantly improve the health status of mice under oxidative damage (Figure 2).

Oxidative stress is known to regulate multiple signaling pathways by activating or inhibiting the activities of various cytokines and enzymes, as well as upregulating or downregulating the levels of receptor ligands, and ultimately affecting gene expression in cells. Accordingly, activities of bone cells associated with bone remodeling were accelerated, such as apoptosis of bone marrow mesenchymal stem cells (BMSCs) and osteoblasts, as well as proliferation and differentiation of osteoclasts, all of which, consequently, resulted in a lag in bone formation rate relative to bone resorption rate [25]. Essentially, oxidative stress induced osteoporosis by disrupting the dynamic balance between the absorption of osteoclasts and the formation of osteoblasts in bone tissue. Osteoporosis has become an epidemic disease affecting public health with diverse etiologies and complex pathogenesis [8]. Among the existing osteoporosis studies, estrogen-deficient ovariectomized (OVX) rats are mainly used as animal models [20]. However, its single etiology has also contributed to limitations on osteoporosis studies. In this study, the combined oxidative stress and low calcium diet-induced osteoporosis was more consistent with the mechanism of clinical osteoporosis involving various factors such as ageing, calcium imbalance, and oxidative stress, as confirmed by our animal experimental results. the micro-CT results showed a decrease in bone mineral density (BMD), bone volume fraction (BV/TV), trabecular number (Tb.N), and trabecular thickness (Tb.Th) (*p* < 0.01 or *p* < 0.05, Figure 5), and an increase in specific bone surface (BS/BV) and trabecular separation (Tb.Sp) (*p* < 0.05) in the model mice, as well as a severe disruption of the bone microstructure (Figure 6B), all indicating the success of this modeling approach.

Bone tissue contains cortical bone distributed on bone surface and cancellous bone distributed on bone interior. Trabecular bone, the extension of cortical bone in cancellous bone, is essentially a multi-aperture reticular structure composed of collagen, minerals, and bone tissue cells that determine bone’s strength and properties [26]. FLL prominently increased BMD to enhance bone mass directly, in addition to heightening BV/TV to improve bone microstructure in mice (*p* < 0.01 or *p* < 0.05, Figure 5). BV/TV is of great value in the evaluation of bone metabolism. When it was elevated, it indicated that bone anabolism was greater than catabolism [27], indirectly reflecting that FLL improved bone metabolism in mice. In addition, at the doses and methods in this study, FLL increased the number and thickness of bone trabeculae and decreased the ratio of bone surface area to bone volume, trabecular separation, and trabecular model factor, although there were no statistical differences. The above results suggested that FLL could, at least partly, strengthen bone microstructure by enhancing bone trabeculae, narrow the porosity by promoting bone anabolism rather than bone catabolism, and improve bone status by inhibiting the transition of bone trabeculae from plate-shaped to role-shaped, thus providing osteoprotective effects.

Expect for bone trabecular, bone health is also related to other components such as periosteum, bone marrow, and blood vessels [28,29]. Abundant marrow fills the space between the marrow cavity and cancellous tissue, providing nutrition for the bones [29]. A high concentration of FLL water extract obviously enhanced the bone wet weight of the femur and humerus in model mice (*p* < 0.01 or *p* < 0.05, Figure 2). Further, FLL upregulated the bone dry–wet ratio of mice, especially in the humerus (*p* < 0.05). Since bone salt accounts for 65–70% of the dry weight of bone and its main component is calcium phosphate [30], FLL might add wet weight by nourishing the bone marrow and add dry weight by accelerating calcification or promoting bone salt precipitation. In addition, in this study, we mainly focused on the effects of FLL on the bone microstructure of mice. It is known that bone structure is divided into diverse aspects such as macroscopic, submicroscopic, microscopic, submicroscopic and nano levels [31]. The drug action of FLL perhaps has other aspects, including altering collagen arrangement and mineral distribution in mice. Clearly, future work should evaluate these hypotheses.

Osteoblasts play an indispensable role in maintaining bone homeostasis against bone resorption in bone remodeling [32]. Our previous studies provided evidence that FLL promoted osteoblastic activity through mitogen-activated protein kinase (MAPK) and Akt signaling pathways [18]. In this study, MC3T3-E1 cells were used as the osteoblast-like cell model. Under the stimulation of H_2_O_2_, the proliferation, differentiation, and OPG protein expression of model cells were notably suppressed, and the RANKL protein secretion was upregulated (*p* < 0.01 or *p* < 0.05, Figure 6), which demonstrated that oxidative stress restrained osteogenesis and accelerated bone resorption. All the FLL-treated groups, but the highest concentration group, showed prominent antioxidant capacity by motivating cell proliferation (*p* < 0.01, Figure 6A). Furthermore, H_2_O_2_ inhibited the osteogenic differentiation of MC3T3-E1 cells by downregulating miR-21 expression [33]. After treatment with FLL, the alkaline phosphatase (ALP) levels were still lower than the H_2_O_2_ group, indicating that FLL prioritized protecting the viability and promoting proliferation in osteoblasts under oxidative damage, rather than initiating early osteoblast differentiation. It also suggested that proliferation and differentiation of osteoblasts were independent physiological activities. The proliferation, differentiation, and migration of osteoblasts are complex, and various second messengers regulate the intercellular interactions [34]. Whether FLL could initiate the differentiation of osteoblasts with a longer treatment time, and then improve ALP activity, is our future research direction.

OPG and RANKL are vital factors secreted by osteoblasts that affect bone metabolism [35]. FLL, in a fluctuating manner, upregulated OPG protein secretion in cell supernatant. At low concentrations (10^−4^–10^−6^ mg/mL), the stimulatory effect of FLL on OPG protein was consistent with that of CCK-8 in the same concentration range, indicating that the OPG secretion was linearly correlated with the number of cells alive at a low concentration. When the concentration of FLL was 1 mg/mL, the cell viability was maximum as well as the OPG protein level was the most outstanding. Contrarily, the trend of the OPG level was opposite to the results of CCK-8 when the concentration of FLL was 10^−1^–10^−3^ mg/mL. That means FLL specifically promotes cell proliferation at high concentrations, while enhancing OPG levels at medium concentrations. The RANKL results showed that all concentrations of FLL could downregulate RANKL expression and there were no significant differences in osteoblasts under oxidative stress (*p* < 0.05, Figure 6D), except at low concentrations (10^−5^–10^−6^ mg/mL). That is, medium and high concentrations of FLL could restrain bone resorption; for another, it accelerated osteogenesis by upregulating the OPG/RANKL ratio. In conclusion, different concentrations of FLL water extracts had various action modes and results on osteoblasts, which provided evidence for the effect of FLL on osteoporosis at the cellular level.

Calcium is also a determinant in bone health apart from oxidative stress and aging [36]. A fecal calcium assay is often used as a part of metabolic balance studies. The 13-week-old mice used in this study were in the growth stage, which absorbed more calcium and excreted less fecal calcium. As compared with the control group, the model mice with low calcium intake still excreted more calcium in their feces (Table 1), indicating that the model group successfully simulated the calcium loss during natural aging. However, after treatment with FLL, the calcium absorption capacity of mice was enhanced and calcium homeostasis was balanced, and therefore fecal Ca was notably lost as compared with the model group (*p* < 0.01). According to the urinary Ca results (Table 1), the actual calcium absorbed into the body and converted to bone in aged mice was the lowest due to aging, despite high calcium intake. Urine Ca/Cr is a marker of bone resorption, and its elevation manifests the occurrence of osteoporosis [37]. The urine Ca/Cr decreased in the mice treated with FLL (*p* < 0.05), which also demonstrated that FLL had anti-osteoporosis activity.

In summary, our in vivo results showed that high concentrations of FLL exhibited prominent anti-bone loss effects in all aspects, while at a medium concentration, FLL showed a preventive effect on fecal Ca, femur index, MDA, and SOD level of the liver and kidney, and the BMD value in osteoporosis mice. The micro-CT images further demonstrated that the protective effect of FLL on bone loss in mice also occurred distinctly from a medium concentration. Up to a high concentration, the bone microstructure of osteoporosis mice was almost restored to that of control mice by FLL. Therefore, the anti-osteoporosis effect of FLL was dose dependent, and a high concentration was the best for short-term usage. However, more research is needed to determine whether the results wouold differ over a more prolonged usage of FLL.

We also analyzed the influence of FLL on aged mice in this study. The results showed that FLL had no therapeutic effect on aged mice with osteoporosis. This was consistent with the traditional Chinese medicine theory of “prevention before disease onset”—treatment with herbs at the beginning of modeling can form a protective effect in the body and reduce the damage during modeling. Since the aged mice had already developed osteoporosis, the bone damage was irreversible. The peak bone mass at the end of growth in the body is decisive for osteoporosis, especially in age-related osteoporosis [38]. If bone quality and strength are optimal at the end of growth, the body can minimize bone loss damage. Therefore, it is vital to develop natural drugs with ideal effects to prevent osteoporosis.

## 5. Conclusions

Our studies indicated that, in progressive osteoporosis, FLL could inhibit oxidative stress by reducing MDA content and enhancing SOD activity; enrich bone nutrition by increasing the bone dry–wet ratio and bone index; improve bone microstructure by regulating bone mineral density, bone volume fraction, and calcium loss; and enhance osteogenesis by promoting cell proliferation and OPG protein expression of osteoblasts. Therefore, FLL might be a promising strategy to prevent osteoporosis and develop natural drugs.

## Figures and Tables

**Table 1:** Effects of *Fructus Ligustri Lucidi* (FLL) on body weight, biochemical parameters, and fecal calcium in female mice.

	Control	Model	FLL-L	FLL-M	FLL-H	Aged
Body Weight Change						
Weight change rate, (%)	3.41 ± 2.84 ^▲▲^	9.34 ± 5.38 **	8.82 ± 2.16 **	6.03 ± 3.36	4.10 ± 2.23 ^▲^	1.63 ± 1.20 ^▲▲^
Serum and Urine Biochemical						
S-Ca, mg/dL	9.81 ± 0.87	10.51 ± 0.56	10.20 ± 0.90	10.09 ± 0.61	10.34 ± 0.94	9.80 ± 1.01
U-Ca, mg/dL	7.29 ± 0.90 ^▲^	8.73 ± 0.80 *	8.50 ± 1.25 *	8.03 ± 1.01	7.69 ± 0.98	6.64 ± 1.28 ^▲▲^
S-Ca/Cr, mg/mg	11.62 ± 2.95	13.92 ± 3.71	13.18 ± 4.02	12.71 ± 2.39	12.20 ± 1.15	13.71 ± 2.71
U-Ca/Cr, mg/mg	0.0936 ± 0.0194 ^▲^	0.1307 ± 0.0375 *	0.1196 ± 0.0359	0.1134 ± 0.0369	0.0961 ± 0.0126 ^▲^	0.1382 ± 0.0319 **
Fecal Calcium Level						
Fecal Ca, mg/g	4.77 ± 1.92 ^▲▲^	13.49 ± 1.68 **	7.85 ± 2.37 *^▲▲^	5.91 ± 2.41 ^▲▲^	4.61 ± 2.58 ^▲▲^	7.23 ± 2.45 ^▲▲^

Note: Mature (13-week-old) Kunming female mice were randomly assigned to five groups (*n* = 8), and 8-month-old female mice (*n* = 8) were assigned to an aged group, with oral administration of FLL or vehicle treatment for 20 d: Control (1.1% dietary Ca); model (0.1% dietary Ca, D-gal injection 10 mg/kg/day); FLL-L (1.75 g/kg/day, model); FLL-M (3.5 g/kg/day, model); FLL-H (7 g/kg/day, model); aged (FLL 3.5 g/kg/d, 1.1% dietary Ca). Data are presented as the mean ± SD. Compared with the control group, ^*^ *p* < 0.05 and ^**^ *p* < 0.01. Compared with the model group, ^▲^ *p* < 0.05 and ^▲▲^ *p* < 0.01.
